# Microcystins in European Noble Crayfish *Astacus astacus* in Lake Steinsfjorden, a *Planktothrix*-Dominated Lake

**DOI:** 10.3390/toxins12050298

**Published:** 2020-05-05

**Authors:** Ingunn Anita Samdal, David Allan Strand, Andreas Ballot, Johannes Christopher Rusch, Sigrid Haande, Kjersti Liv Eriksen Løvberg, Christopher Owen Miles, Trude Vrålstad

**Affiliations:** 1 Norwegian Veterinary Institute, P.O. Box 750 Sentrum, 0106 Oslo, Norway; david.strand@vetinst.no (D.A.S.); johannes.rusch@vetinst.no (J.C.R.); kjersti.lovberg@vetinst.no (K.L.E.L.); Christopher.Miles@nrc-cnrc.gc.ca (C.O.M.); trude.vralstad@vetinst.no (T.V.); 2 Norwegian Institute for Water Research, Gaustadalléen 21, 0349 Oslo, Norway; andreas.ballot@niva.no (A.B.); sigrid.haande@niva.no (S.H.); 3 Department of Biosciences, University of Oslo, P.O. Box 1066, Blindern, NO-0316 Oslo, Norway; 4 National Research Council Canada, Halifax, NS B3H 3Z1, Canada

**Keywords:** *Astacus astacus*, cyanobacteria, ELISA, noble crayfish, food safety, microcystin, *Planktothrix*, qPCR, tolerable daily intake, Steinsfjorden, Norway

## Abstract

Lake Steinsfjorden, an important Norwegian location for noble crayfish (*Astacus astacus*), is often affected by cyanobacterial blooms caused by microcystin (MC)-producing *Planktothrix* spp. The impact of MCs on noble crayfish as a food source and crayfish health is largely unknown. We investigated the quantities and correlations of MCs in noble crayfish and lake water during and after a cyanobacterial bloom peaking in June–July 2015. Noble crayfish and water samples were collected monthly from June to October 2015 and in October 2016. The content of MCs was analysed by ELISA from tail muscle, intestine, stomach and hepatopancreas. PCR analysis for *Planktothrix* gene markers was performed on crayfish stomach content. Water samples were analysed for phytoplankton composition, biomass and MCs. PCR-positive stomach contents indicated *Planktothrix* to be part of the noble crayfish diet. Concentrations of MCs were highest in the hepatopancreas, stomach and intestine, peaking in August–September. Tail muscle contained low concentrations of MCs. Similar levels of MCs were found in crayfish from 2016. Except in September 2015, a normal portion of boiled noble crayfish tails was below the tolerable daily intake (TDI) for MCs for humans. Removing the intestine more than halved the content of MCs and seems a reasonable precautionary measure for noble crayfish consumers.

## 1. Introduction

**Figure 1:**
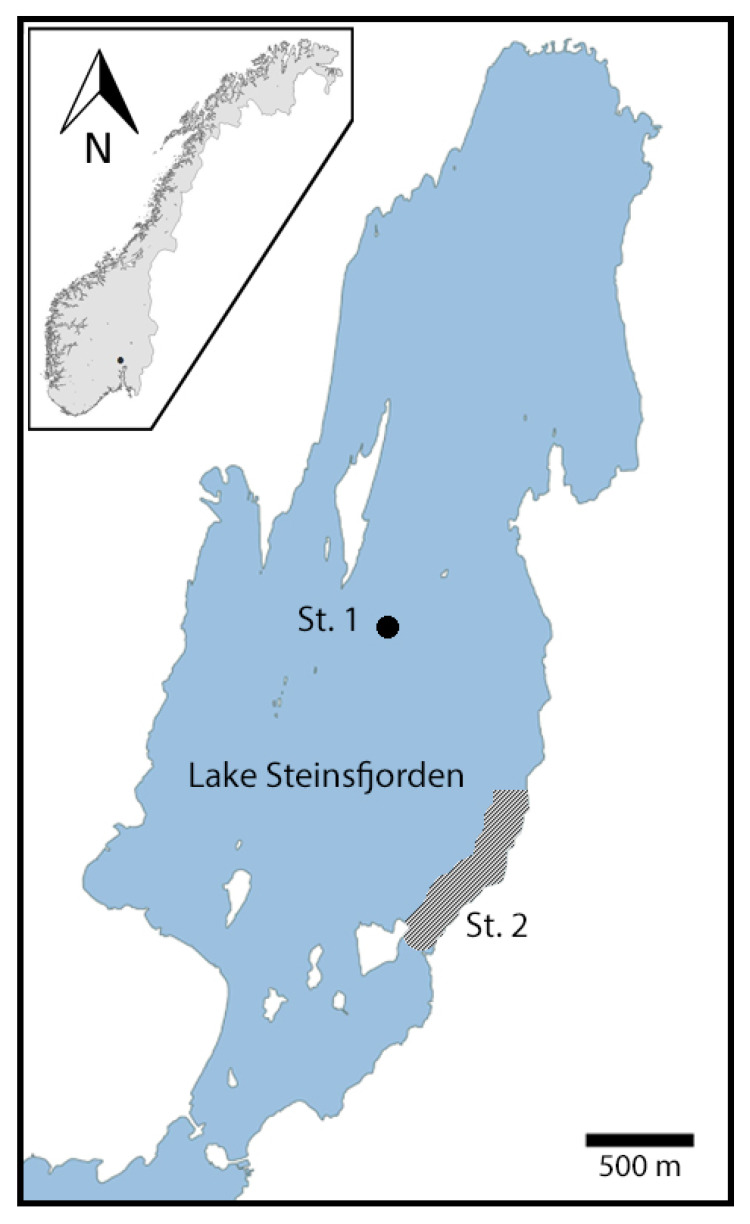
Map of Lake Steinsfjorden. In the south, the lake is connected through a narrow shallow passage with a low water exchange to the larger and deeper Lake Tyrifjorden. The map shows the locations of the water sampling (Station 1 (St. 1) at 60.09452°N, 10.32427°E) and the noble crayfish sampling (shaded area of the east shore, St. 2). The location of L. Steinsfjorden in Norway is shown in the inset. Kartverket.no

Lake Steinsfjorden is one of the most important Norwegian locations for the red-listed noble crayfish (*Astacus astacus*) [1]. This crayfish species native to Europe has severely declined over the past decades, and is now classified as “vulnerable” on the IUCN red list [2] and ”endangered” on the Norwegian red list [3]. However, the great cultural and economic interest in the noble crayfish as a delicacy for human consumption is a strong driver for its protection in Norway. Thus, the Norwegian legislation and management of noble crayfish allows for a strictly regulated fishery [4]. Lake Steinsfjorden accounts for approximately 25–30% of the annual harvest in Norway [1,5,6]. The lake is also used for agricultural irrigation and recreational purposes, such as fishing, swimming and water sports. The dimictic and mesotrophic lake has a surface area of 13.9 km^2^ and a maximum depth of 24 m [7]. The lake is located in southeastern Norway (Figure 1) and is connected to the larger and deeper Lake Tyrifjorden through a narrow shallow passage with a low water exchange [7].

**Figure 2:**
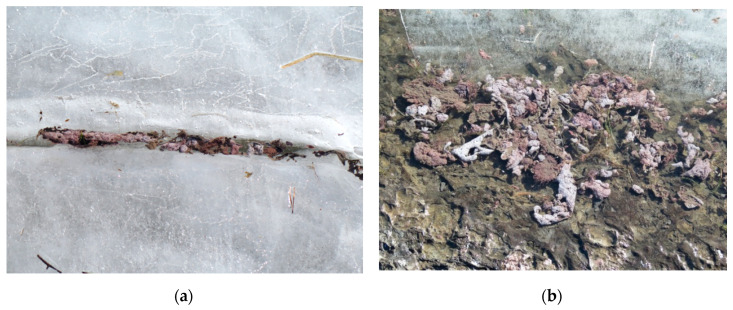
Photographs of *Planktothrix* clumps from Lake Steinsfjorden in the March 2015: (**a**) in a crack in the ice, and; (**b**) on the shoreline, illustrating the large quantities of *Planktothrix* biomass in the ecosystem.

For decades, L. Steinsfjorden has experienced regular cyanobacterial blooms, usually *Planktothrix agardhii* and *Planktothrix rubescens* [7,8], both known microcystin (MC) producers. *Planktothrix* spp. are known to form metalimnetic blooms during the growth season in mesotrophic lakes like L. Steinsfjorden [7,9]. Metalimnetic blooms at 10–12 meters depth are more common than surface blooms in this lake. During the autumnal circulation the *Planktothrix* filaments become evenly distributed in the whole water body of L. Steinsfjorden and can even survive in large biomasses under the ice cover during winter. In some years after the ice melt, large quantities of viable *Planktothrix* filaments can be observed in shallow areas and piling up on the shores (Figure 2) [10]. The accumulated *Planktothrix* biomass is a potential food source for the omnivorous noble crayfish and could therefore lead to an uptake of MCs in crayfish.

MCs are toxic cyclic heptapeptides that usually contain the unusual β-amino acid 3-amino-9-methoxy-2,6,8-trimethyl-10-phenyl-4,6-decadienoic acid (Adda). So far, at least 279 MC analogues have been reported [11], most commonly MC-LR, -LA and -RR. Although none of these three variants have been reported from L. Steinsfjorden, 17 other MC variants have been reported in water samples or *Planktothrix* cultures isolated from the lake [12].

Decapods, including crayfish and freshwater shrimps, have been found to accumulate MCs in various organs, especially the hepatopancreas and gonads [13]. They may be able to conjugate MCs, as elevated levels of glutathione *S*-transferase can be found in contaminated crabs [14]. Decapod health effects of MCs are not well studied, but mortalities of white shrimp have been reported in Texas aquaculture ponds under blooms dominated by *Microcystis aeruginosa* and *Anabaena* spp., leading to the accumulation of 55 μg/g MC-LR in the shrimp hepatopancreas, but with a toxin concentration below 0.1 μg/g in the tail muscle [13]. It has been shown for North-American signal crayfish (*Pacifastacus leniusculus*) that when fed on toxic cyanobacteria, MCs are accumulated in their hepatopancreas without apparent negative influence on the crayfish health [15]. However, the 14-day duration of the experiment may not be sufficient to answer this question. The accumulation of MCs may vary in different decapod species, and red swamp crayfish (*Procambarus clarkii*) was found to accumulate considerable MC concentrations in the intestine [16]. Only a few studies address the presence of MCs in the noble crayfish [12,17,18]. One report suggested that noble crayfish in L. Steinsfjorden might take up and retain microcystins [19] and a subsequent study confirmed the presence of MCs in 10 noble crayfish from L. Steinsfjorden [12], suggesting that a more comprehensive study should be performed.

MCs constitute a hazard to humans when these toxins enter drinking water sources, or into the food chain in the form of contaminated edible aquatic animals such as fish and decapods [13]. To protect consumers from the adverse effects of MCs, the World Health Organisation (WHO) has proposed a provisional upper limit in drinking water of 1 µg/L for MC-LR and a tolerably daily intake (TDI) of 0.04 µg/kg [20]. However, it is unclear whether MCs pose a risk to crayfish health, or to the vertebrates and human consumers that eat crayfish from lakes affected by cyanobacterial blooms. In the Barataria estuary system of southeastern Louisiana, high concentrations of MCs were found in the estuarine blue crab (*Callinectes sapidus*) living in the hyper-eutrophic freshwater lake Lac des Allemands. Here, the highest tissue concentrations of MCs were detected in the hepatopancreas with 820 μg/kg, and 105 μg/kg in the edible muscle [21]. A meal of these animals would clearly exceed the TDI guideline.

Since L. Steinsfjorden is an important noble crayfish locality that accounts for a substantial part of the annual noble crayfish harvest in Norway, the aim was to study the degree of uptake of MCs by the crayfish and the distribution of these toxins in the different crayfish tissues. The aim was also to evaluate any potential health risks to humans associated with consumption of noble crayfish from this lake, using the same TDI recommendations as for drinking water. Information on the levels of MCs in selected organs and edible parts of noble crayfish from this exposed, but stable, crayfish population could also shed new light on the dynamics of MC uptake and depuration.

## 2. Results and Discussion

### 2.1. Cyanobacteria and MCs in Lake Steinsfjorden

**Figure 3:**
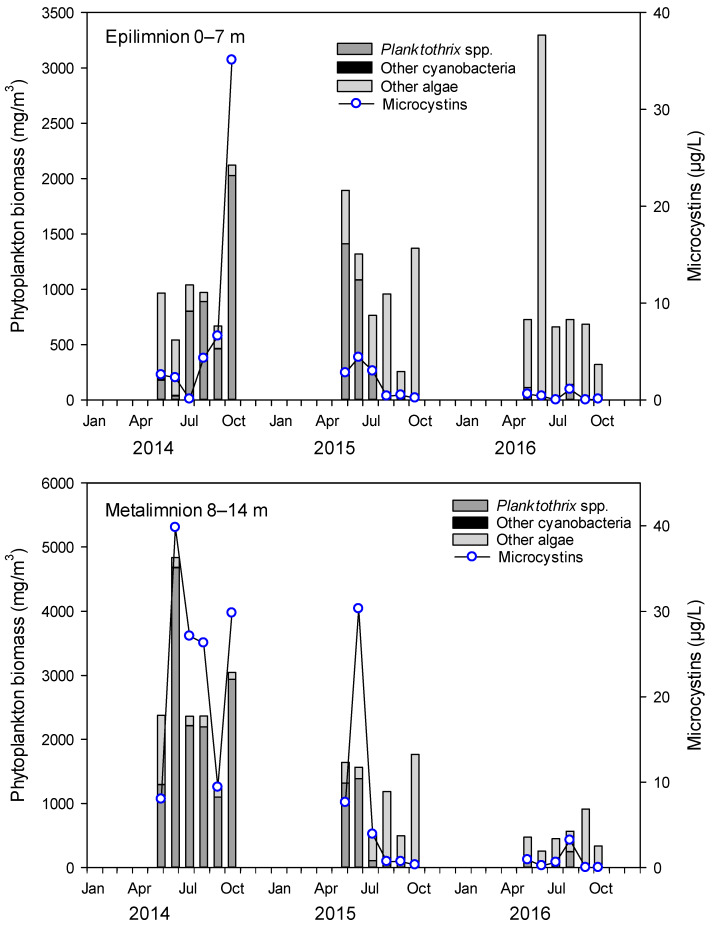
Phytoplankton biomass, and microcystin (MC) concentrations by Adda-ELISA, in Lake Steinsfjorden for 2014–2016 in the epilimnion (**top**) and metalimnion (**bottom**).

In 2014 and 2015, L. Steinsfjorden (Figure 1) had relatively high *Planktothrix* biomasses (up to 2.0 mg/L in the epilimnion (0–7 m) and 4.7 mg/L in the metalimnion (8–14 m) at St. 1 (Figure 1), when compared to years before and after (Figure S1). During the winter of 2014–2015, *Planktothrix* spp. survived under the ice. After the ice melted in April 2015, large quantities of *Planktothrix* biomass were observed in shallow areas and piled up on the shore (Figure 2). In summer 2015, the phytoplankton was dominated by *Planktothrix* spp. in May–July and by diatoms (*Bacillariopyceae*) in August–October (Figure 3). In 2014, MC concentrations by Adda-ELISA increased to 35 µg/L in October in the epilimnion, whereas high levels up to 40 µg/L were measured in the metalimnion in June, with around 30 µg/L in July, August and October (Figure 3). In 2015, the MC concentrations by Adda-ELISA were 30 µg/L in the metalimnion in June 2015 and between 0.5–4.0 µg/L in both epilimnion and metalimnion for the rest of summer 2015. There was a significant correlation (0.80, *p* < 0.0001) between the biomass of cyanobacteria and microcystins in the water for 2014–2016 (Figure S2). Compared to the study of MC accumulation in red swamp crayfish (*Procambarus clarkii*) [16], where the water contained 1.76 µg/L MCs at the crayfish harvesting location, our study site had much higher MC concentrations in the early summer, but similar concentrations during crayfish sampling from July 2015 and onwards.

In spring 2015, the *Planktothrix* clumps were likely to be a food source for noble crayfish living in the shallow areas of L. Steinsfjorden. The MCs produced by *Planktothrix* would then have been ingested together with the *Planktothrix* biomass. The annual monitoring of MCs at different bathing places [22,23] and at the centre of L. Steinsfjorden showed, however, that concentrations up to 40 µg/L can be observed in years with *Planktothrix* blooms (Figure 3). The *Planktothrix* biomass and MC values measured in the surrounding waters clearly support the occasional exposure of noble crayfish to MCs.

We were not able to directly measure any health effects on the noble crayfish from the lake. However, the lack of observed mortalities combined with data from the yearly monitoring of noble crayfish indicates that adult noble crayfish exhibit tolerance to exposure to MCs. Although lower than in 2014 and 2016, the yearly monitoring did not reveal any dramatic reduction in the population density of noble crayfish in L. Steinsfjorden in 2015, and trapping data from the past 15 years suggest a relatively stable population density of noble crayfish in the lake with a promising increase in 2017–18 [24]. An experimental study on juvenile red swamp crayfish showed that they also exhibited tolerance to MCs [25]. We have no data for noble crayfish juveniles in L. Steinsfjorden, but the long-term stability in the noble crayfish population density suggests that the frequent cyanobacterial blooms in the lake likely have no detrimental impact on the crayfish juveniles.

### 2.2. Planktothrix in the Crayfish Diet

PCR analysis of the stomach tissue/contents (Table 1) indicated that filaments of *Planktothrix* were part of the diet of noble crayfish from Lake Steinsfjorden. The stomach contents of all five analysed noble crayfish caught in June 2015 were positive for *Planktothrix* DNA using the phycocyanin gene intergenic spacer cpcB-cpcA (PC-IGS) as a marker (Table 1), whereas only three of the five examined noble crayfish caught in September 2015 were positive. The winter of 2014–15 was also characterized by large *Planktothrix* biomasses accumulating under the ice, suggesting the possible ingestion of *Planktothrix* filaments and MCs by noble crayfish in spring 2015 (Figure 2). It is not known if noble crayfish have developed any specific preferences for avoiding or selecting toxic *Planktothrix* in their diet, but it has been shown that red swamp crayfish preferred eating MC-producing strains of *Microcystis* over non-MC-producing strains, and showed improved growth when eating the MC-producing strains [25]. In that study, juvenile red swamp crayfish tolerated MC-producing cyanobacteria better than the non-MC-producing strains, and experimental juvenile crayfish accumulated up to 2900 ng/g of MCs (dry weight). These crayfish nonetheless showed improved growth, lipid stores, and higher protein levels compared to control groups and crayfish fed non-MC-producing *Microcystis* strains [25]. In our study, the ten noble crayfish caught in October 2016 contained no traces of *Planktothrix*-specific markers in the stomach. This corresponds well to the low level of *Planktothrix* biomass observed in 2016, even though elevated levels of MCs were present in noble crayfish tissues—including the stomach—at this time (Table 1). All stomach content samples analysed were positive for the noble-crayfish-specific cytochrome oxidase subunit I gene (COI) (Table 1), excluding the possibility of a false-negative result/type II error due to failed PCR reaction.

### 2.3. MCs in Crayfish Tissues

Prior to the analysis of noble crayfish from L. Steinsfjorden, control crayfish from the Kasa crayfish farm at Hvaler were investigated with the multihapten MC-ELISA to assess the influence of possible matrix effects from the various tissues on the ELISA. For tail muscle tissue, matrix effects were minor even at 10-fold dilution, whereas a 100-fold dilution was required to overcome matrix effects for hepatopancreas and intestine (Figure S3). However, due to the sensitivity of the assay, most of the tissue extracts from crayfish from L. Steinsfjorden had to be diluted more than this to adjust them to within the working range of the ELISA. No MCs were detected in tissues from pooled samples of raw or cooked control crayfish, nor in any of the samples used for matrix effect evaluation.

MCs were detected with the multihapten MC-ELISA in all 110 individuals of noble crayfish collected from L. Steinsfjorden in 2015 and 2016, with high individual variation. A linear mixed effect (lme) model was used to investigate whether the treatment (raw versus boiled), gender, length of the noble crayfish, tissue type, month or monthly exposure to MCs from the water influenced the observed MC-levels in the noble crayfish. The selected model (based on the Akaike’s information criterion) that best described the MC concentration (ng/g) in tissue per crayfish *i* and replicate *j* was found to be:

(1) $$\log_{10}MCs\;ng/g\sim \;\beta 0+\beta 1Month{\left(October\_2015\;\right)}_{ij}\cdot \;\beta 2Tissue_{ij}+a_{i}+\varepsilon_{ij}$$

Random intercepts of individual crayfish (*a_i_*) and residuals (*ε_ij_*) were assumed independent and normally distributed around zero mean. The standard deviation for residuals (n = 440) and random effects of crayfish (n = 110) were 0.43 and 0.44, respectively. The model only includes the significant factors, which were found to be tissue and an interaction with the month of October. These factors are summarized in Table 2.

**Figure 4:**
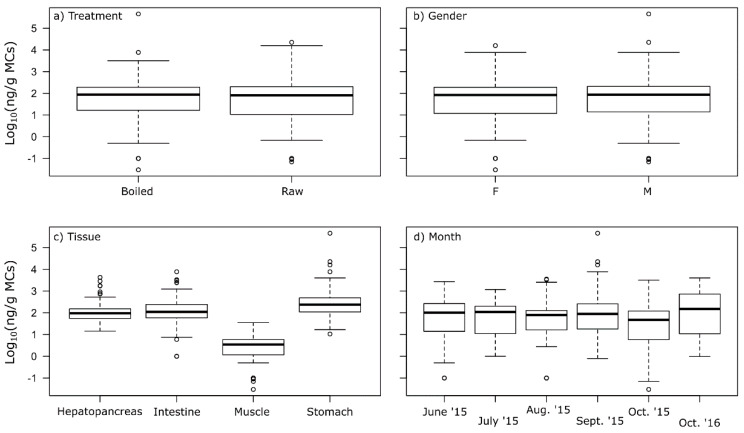
Boxplot of Log_10_ MC concentration (ng/g) by ELISA in noble crayfish *A. astacus*; (**a**) boiled (n = 50) versus raw (n = 50) for 2015; (**b**) gender, i.e., female (n = 42) or male (n = 58); (**c**) type of tissue, i.e., hepatopancreas, tail muscle, stomach, and intestine (n = 100) from 2015; (**d**) hepatopancreas, tail muscle, stomach, and intestine combined, by month from June to October 2015 and in October 2016 (n = 20 for each month, apart from n = 10 for Oct 2016). The thick lines are median values, the boxes indicate 25 and 75 quartiles of the data set, and the bars extend to min/max values of MC concentrations in the tissues. Observations shown as circles are regarded statistically as outliers.

There was no significant difference in the levels of MCs between the raw and boiled crayfish (Figure 4a), nor between female and male crayfish (Figure 4b). Neither the total length of the crayfish nor the monthly toxin concentration in the lake contributed to explaining the MC concentrations in the crayfish tissues (Figures S4 and S5). Although not directly comparable, Tricarico et al. [16] analysed the MCs in naturally contaminated red swamp crayfish by ELISA (EnviroGard Microcystins Plate Kit) and found that females accumulated significantly higher amounts of MCs in their hepatopancreas than males. Furthermore, small individuals of red swamp crayfish contained significantly higher concentrations of MCs than large individuals. In our study, crayfish size ranged from 75 to 109 mm total length, with a mean of 89.1 mm, and would all classify as large individuals.

Analysis of MC concentrations in the hepatopancreas (liver), intestine, tail muscle and stomach, showed large individual variations within tissue type (Figure 4c), and significant differences between tissue types (Table 2). The stomach of the crayfish contained significantly higher levels of MCs (median 220 µg/kg and mean 5430 µg/kg) than the hepatopancreas (median 91 µg/kg and mean 199 µg/kg) and intestine (median 108 µg/kg and mean 329 µg/kg), whereas tail muscle contained much lower concentrations of MCs (median 3.6 µg/kg and mean 5.1 µg/kg) (Table 2 and Table 3). For all tissues, the mean was higher than the median, and this was especially the case for samples of the stomach where the mean was 25 times higher than the median value, indicating a skewed distribution due to some individuals with very high MC concentrations in their stomach. Our results are consistent with earlier studies of freshwater crayfish and MC-producing cyanobacteria, where high levels of MCs were reported in the hepatopancreas, stomach and intestine, but remained low in the edible tail muscle [13,16,25]. However, Tricarico et al. [16] found by far the highest levels of MCs in the intestine, while we found the highest levels in the stomach. For crabs, higher levels of MCs have been reported in the edible muscle than so far observed for crayfish. For example, crabs in Sepetiba Bay (Rio de Janeiro) had up to 103 μg/kg MCs in the edible muscle [26], and blue crabs in the Barataria estuary (southeastern Louisiana) had up to 105 μg/kg MCs in muscle [21].

The month-to-month variations for all four tissues combined (Figure 4d) showed relatively stable MC concentrations in the crayfish during 2015. For comparison, 10 noble crayfish samples from October 2016 were collected and analysed, and, surprisingly, these contained MCs at similar levels, even though only low levels of *Planktothrix* were observed in the lake throughout the summer of 2016 (Figure 3).

**Figure 5:**
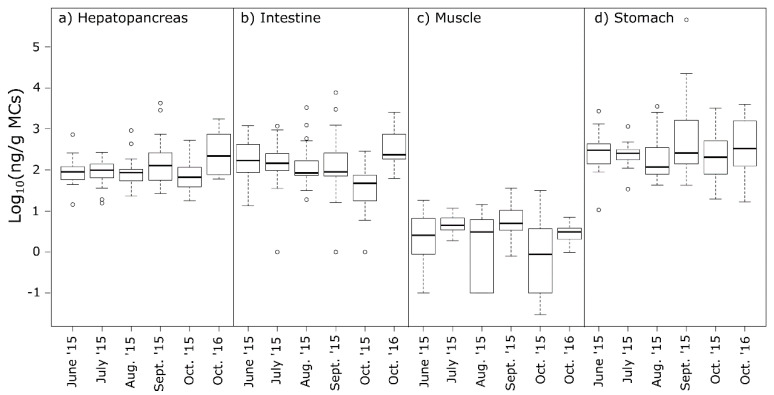
Boxplot of Log_10_ MC concentration (ng/g) by ELISA in the four tissues; (**a**) hepatopancreas; (**b**) intestine; (**c**) tail muscle; (**d**) stomach, for 110 noble crayfish (n = 20 for each month, except for Oct 2016 where n = 10). Dark lines are median values, the boxes indicate 25 and 75 quartiles of the data set, and the bars extend to min/max values of MC concentrations in the tissues. Observations shown as circles are regarded statistically as outliers, although here due to large individual variation.

The month-to-month variations for each individual tissue (Figure 5) showed a similar pattern to the combined tissues. Levels of MCs in the stomach were significantly higher than in the hepatopancreas and intestine, whereas levels in the tail muscle were significantly lower during all months, although there was high variation between individuals. The lme model revealed a significant interaction between “October month” and “tissue type” (Table 2), showing that there were significantly lower concentrations in the tail muscle and intestine in samples from October 2015, compared to the other months (Figure 5, Table 2 and Table 3). Although there were lower levels of MCs in the lake water in 2016 than in 2015, there was no significant difference in the toxin concentrations in the crayfish tissues between October 2016 and 2015. This is puzzling, since the bloom of toxic *Planktothrix* began to decline in July 2015. Other studies have shown that a depuration time of 3 weeks significantly reduced the levels of MCs in the muscle, but not in the intestine [16]. Since the absence of *Planktothrix* in the water did not lead to diminished levels of MCs in the studied crayfish organs, it seems likely that the crayfish either release bound/accumulated MCs for a long period after exposure, or feed on sources in which MCs have accumulated (e.g., dead fish, zooplankton etc.). Alternatively, other sources than *Planktothrix* may produce MCs that enter the crayfish diet, although significant amounts of other cyanobacterial genera were not observed in the water during the study.

**Figure 6:**
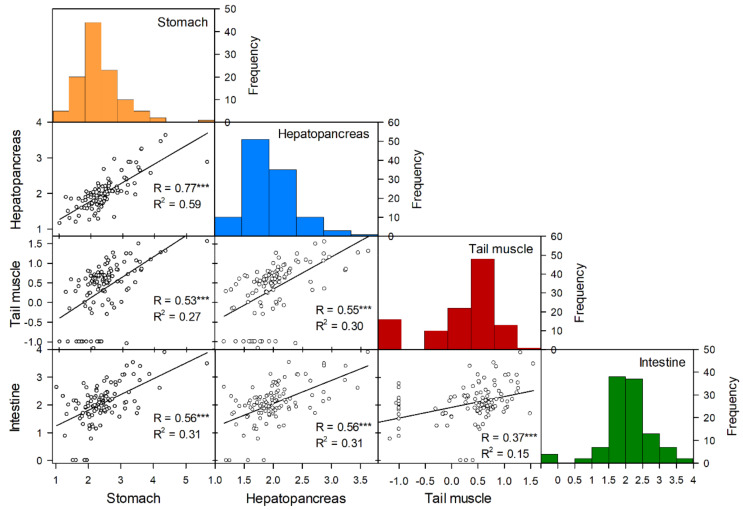
Correlation matrix showing pairwise correlations (Pearson’s rank) of Log_10_ MC concentration (ng/g) by ELISA in the four tissues hepatopancreas, tail muscle, stomach, and intestine from individual crayfish (n = 110). The lower left area shows bivariate scatterplots of individual samples with a fitted line, the coefficient of correlation R-values and the coefficient of determination R^2^-values and the significance level (asterisks). The asterisks (***) indicate significance level *p* < 0.001.

The Pearson rank test revealed a significant correlation between MC concentrations in the four tissue types (Figure 6). The correlation (R^2^) appeared to be higher between tissues directly related to the digestive system of the crayfish, whereas the correlation between muscle tissue and the digestive system seemed lower.

### 2.4. Food Safety

The WHO provisional TDI of MCs for humans is 0.04 µg/kg/day [20]. For a 60 kg person, that corresponds to 2.4 µg/day. The edible part of the noble crayfish consists of the tail muscle with its encapsulated intestine. In this study, the average tail muscle weighed ~4 g and the intestine constituted ~1/30 of the tail muscle by weight. Thus, 1 kg crayfish corresponds to a meal of ~100 g of tail muscle containing 3.3 g of intestine. A normal portion of boiled whole noble crayfish is about 1 kg, which corresponds to ~25 whole noble crayfish of 9–10 cm. The edible parts (tail muscle including the intestine) contained levels of MCs measured by multihapten-ELISA up to the TDI in August (Figure S6) and above the TDI in September 2015 (Figure S7). Table 3 shows how much tail muscle tissue with, and without, intestine would need to be consumed to reach the MC TDI for Jun–Oct 2015. In August 2015, an average of 39 tails with intestine would expose the consumer to the TDI, whereas if the intestines were removed then 157 noble crayfish tails could be consumed before reaching the TDI. In either case, this would be significantly more than the portion size considered normal (25 noble crayfish). Despite the intestine being small compared to the tail muscle, these results show that the intestine contributes substantially to the total amount of MCs consumed. Removal of the intestine more than halves the total MC content of the edible part, and therefore seems to be a reasonable food safety precaution when consuming noble crayfish from cyanobacteria-dominated waterbodies.

The levels of MCs measured by multihapten-ELISA in the noble crayfish from October 2016, after a summer with very low concentrations of cyanobacteria in the epilimnion and metalimnion in comparison to 2015, show that the cyanobacterial levels in the water column and MC levels in the water are not a good indicator for predicting levels of MCs in noble crayfish in L. Steinsfjorden. As no samples were available from November 2015 to April 2016, little is known about the situation in the lake during this time, although no bloom was observed under the ice that winter.

The absence of a cyanobacterial bloom in summer 2016 and the lack of *Planktothrix* spp. specific genes from the noble crayfish stomach indicate a different source of microcystins than the monitored *Planktothrix* spp. Therefore, the noble crayfish must have obtained the microcystins from a source other than *Planktothrix* or have retained bioaccumulated microcystins from an earlier exposure. Since noble crayfish are omnivorous, they most likely prey on other organisms that have been grazing on cyanobacteria or organisms that have preyed on other organisms that have been grazing on cyanobacteria, as well as consuming clumps of cyanobacteria themselves, when present. Gaget, et al. [27] described microcystin production by two benthic cyanobacteria (*Nostoc linckia* and *Limnothrix mirabilis*) in an Australian reservoir and Cantoral Uriza, et al. [28] described MC production in Spanish benthic cyanobacterial strains from different habitats, so the presence of benthic cyanobacteria may be something to investigate in relation to MCs in crayfish in L. Steinsfjorden.

## 3. Conclusions

Lake Steinsfjorden experienced a bloom of *Planktothrix* in 2014 and 2015 and microcystins were found in the lake water. Noble crayfish from the lake had MCs in all four investigated tissues (intestine, stomach, hepatopancreas and tail muscle) and 8 of 10 tested stomach samples contained markers for *Planktothrix* spp., showing the cyanobacterium to be part of the noble crayfish diet. No unexpected mortalities were observed, and circumstantial evidence suggests a high tolerance to MCs in noble crayfish. Edible parts (tail muscle plus encapsulated intestine) contained low levels of MCs, just above the WHO TDI in September 2015. Removing the intestine from the tail reduced the MC content of the edible parts by 2–4-fold, to well below the TDI.

The finding of MCs at elevated levels in October 2016 more than one year after the *Planktothrix* bloom might indicate very slow depuration of MCs in the crayfish tissues and/or new supply through bioaccumulation in the food web, but also calls for more studies of alternative MCs sources in the lake. The results further suggest that surveillance of *Planktothrix* spp. and MCs in the lake water is unsuitable for predicting MC levels in noble crayfish in L. Steinsfjorden.

## 4. Materials and Methods

### 4.1. Materials

Inorganic chemicals and organic solvents were reagent grade or better. Standard of MC-LR for the multihapten-ELISA were from Enzo Life Sciences Inc. (Farmingdale, NY, USA), and this was in-house calibrated against a CRM-MCLR from NRC (Halifax, NS, Canada). The standard for the Adda-ELISA was as provided with the kit (Abraxis LLC, Warminister, PA, USA).

### 4.2. Water samples

Integrated water samples for phytoplankton composition and biomass and for MC analyses were taken monthly from May–October in 2014, 2015 and 2016 at the deepest part of the lake (St. 1; 60.09452°N, 10.32427°E) (Figure 1) from epilimnion (0–7 m) and metalimnion (8–14 m) with a 2-m Ramberg sampler. For quantitative and qualitative phytoplankton analyses, 100 mL water samples were fixed with acidic Lugol’s solution and stored in the dark until further analysis. For MC analyses, 5 mL water samples were frozen at −20 °C and stored until further analysis.

### 4.3. Phytoplankton

Phytoplankton taxa were counted in sedimentation chambers (Hydro-Bios Apparatebau GmbH Kiel, Germany) using an inverted microscope (Leica DMi8, Leica Microcystems, Wetzlar, Germany) according to Utermöhl [29]. Phytoplankton was determined using standard key literature [30,31,32,33]. The specific density of phytoplankton cells was calculated to be 1 g/cm^3^.

### 4.4. Noble Crayfish (A. astacus)

Wild noble crayfish were caught using baited traps by a local landowner with permission from the county governor of Buskerud. Once a month, from June to October 2015, 30 baited traps were put out in the evening on the eastern shore of the lake and hauled in the next morning (Figure 1). Twenty noble crayfish were randomly selected from each catch, of which 10 were frozen directly (raw crayfish) and ten were boiled and then frozen (boiled crayfish). The bait used for trapping was locally caught fish to avoid accidental transfer of pathogens into the lake. Another 10 noble crayfish were randomly selected from the catch from the same location in October 2016 and frozen raw (raw crayfish). A total of 100 noble crayfish (42 female, 58 male) with an average size of 88.1 mm total length (SD 68 mm) were thus analysed from 2015 and a total of 10 noble crayfish (4 female, 6 male) with an average size of 99 mm total length (SD 5.5 mm) were analysed from 2016. Control noble crayfish were obtained from an aquaculture facility (Kasa crayfish) at Hvaler.

The noble crayfish were dissected in a partly-thawed condition, using clean equipment for each animal. Tissue samples were taken from the stomach, hepatopancreas, intestine and abdominal tail muscle and stored in separate 50 mL tubes. The tissue samples were weighed and subjected to two freeze-thaw cycles in order to induce cell rupture, followed by addition of 100% methanol (1:9 w/v) and homogenization with an Ultra-turrax blender. Aliquots of the homogenate were filtered and stored at −20 °C until analysis.

### 4.5. Adda-ELISA

The water samples from the sampling point St. 1 in L. Steinsfjorden (Figure 1) were tested for MCs using the Abraxis microcystin-Adda-ELISA (Abraxis LLC, Warminister, USA) following the manufacturer’s instructions. The test is an indirect competitive ELISA based on the recognition by specific antibodies of the Adda moiety found in most MCs and nodularins. Ready-made standards of MC were provided at 0, 0.15, 0.40, 1.0, 2.0 and 5.0 ppb (i.e. ng/mL). Before analysis, 5 mL of each water sample was frozen and thawed three times to release the toxins. The color intensity of the ELISA test was evaluated by absorbance at 450 nm on a PerkinElmer 1420 Multilabel counter Victor^3^ (PerkinElmer, Waltham, MA, USA), and concentrations were determined by fitting to a linear curve according to the manufacturer’s instructions.

### 4.6. Multihapten-ELISA

The concentration of MCs in each noble crayfish extract was determined by indirect competitive ELISA as described by Samdal, et al. [34] based on the multihapten approach, with only minor adjustments to plate-coater and antibody concentrations to optimize the assay. Optimal concentrations of plate-coating antigen (2 μg/mL), antiserum 80289-5b (1:20,000), and rabbit antisheep IgG−HRP (1:8000) from Santa Cruz Biotechnology (Dallas, TX, USA) were determined by checkerboard titrations followed by optimization of the standard curve. The MC-LR standard in methanol (500 ng/mL) was diluted in PBST to give a methanol concentration of 10%, and then in a threefold dilution series in sample buffer, giving standard concentrations of 50, 16.7, 5.56, 0.62, 0.20, 0.069, 0.023, 0.0076, and 0.0025 ng/mL. Serial dilutions of standards and samples were performed in duplicate. All incubations were performed at ~20 °C. Absorbances were measured at 450 nm using a SpectraMax i3x plate reader (Molecular Devices, Sunnyvale, CA, USA). Assay standard curves were calculated using 4-parameter logistic treatment of the data using SoftMax Pro version 6.5.1. (Molecular Devices, Sunnyvale, CA, USA).The assay working range was defined as the linear region at 20−80% of maximum absorbance (A_max_). Minor matrix effects were observed for the noble crayfish tissue samples, but these were abolished at 100-fold dilution for hepatopancreas, stomach and intestine and at 10-fold dilution for the tail muscle. An initial experiment was done on three cooked and three uncooked farmed crayfish (Kasa crayfish, Hvaler, Norway). These were dissected separately and the tissue types pooled separately for the cooked and uncooked samples. No microcystins were detected in any of the pooled tissues in the cooked or uncooked controls.

### 4.7. DNA Isolation

The methanolic noble crayfish stomach tissue homogenates were used for isolating DNA. 1 mL of the stomach tissue homogenized in methanol was transferred to a 2 mL tube. The tube was centrifuged to pellet the tissue and then vacuum dried to evaporate the methanol using a Savant DNA 120 Speedvac concentrator (Thermo Fisher Scientific, Waltham, MA, USA). DNA was isolated from the tissue pellet and its methanolic residue (*A. astacus* stomach) using the Qiagen DNeasy Blood and Tissue kit (Qiagen, Hilden, Germany) according to the manufacturer’s protocol. DNA was isolated from stomach tissue from a total of 20 noble crayfish.

### 4.8. PCR

Real-time PCR primers and protocols were developed for the detection of DNA of *Planktothrix* spp. in the stomach and gut contents of the noble crayfish using the phycocyanin operon (cpcB-cpcA intergenic spacer PC-IGS) of *Planktothrix* spp. Specific primers were developed in this study using FastPCR [35] amplifying a 105 bp sequence (forward primer (PLcpc4f): 5′-ATG AAA ACC CCC CTG ACT G-3′ and reverse primer (PLcpc4r): 5´-GCT TTG GCT TGA CGG AAA C-3´). Real-time PCRs for the selected PC-IGS were performed on a Bio-Rad CFX96 Real-Time PCR Detection System (Bio-Rad, Hercules, CA, USA) using SsoFast EvaGreen Supermix (Bio-Rad). PC-IGS fragments were amplified using a cycling protocol comprising of one cycle of 30 s at 95 °C, and then 40 cycles each consisting of 5 s at 95 °C, 15 s at 60 °C, and 10 s at 72 °C, followed by a melting curve analysis (65–95 °C). As a control for DNA presence, all samples were additionally tested with the *Astacus*-specific primers amplifying a 65 bp sequence (forward primer (Astast_COI_F0336): 5´-GAT TAG AGG AAT AGT AGA GAG-3´ and reverse primer (Astast_COI_R0397): 5´-CTG ATG CTA AAG GGG GAT AA-3´) [36] for one cycle of 30 s at 95 °C, and then 40 cycles each consisting of 5 s at 95 °C, 15 s at 56 °C, and 10 s at 72 °C, followed by a melting curve analysis. (65–95 °C).

### 4.9. Statistics

The MC concentrations were calculated per gram tissue (ng/g) for each sample, then log_10_-transformed for all statistical analyses. Statistical analyses were performed in Rstudio version 1.1.456 [37] using R version 3.5.2 (Vienna, Austria) [38]. A Pearson’s rank correlation test was used to compare the MC content of the different tissues. We also tested the correlation between MC concentration in lake water and cyanobacterial biomass using the Pearson’s rank correlation test. As four tissues were sampled per noble crayfish, an lme model was used to account for the dependence structure in the data set. The R package “nlme” was used for the linear mixed-effects models [39]. We set “Noble crayfish” as the random factor while we included the following fixed factors in the model to describe the MC concentrations in the tissue: “Noble crayfish total length”, “Gender”, “Month”, “Monthly toxin concentration in the lake”, “Treatment (Boiled or Raw)” and “Tissue type”. We selected the best model describing the dataset based on the Akaike’s information criterion-value [40].

### 4.10. TDI Calculations

The weight of tail muscle needed to be consumed to achieve the TDI was calculated using the formula:

(2) $$y=\frac{TDI\cdot Weight\cdot 1000}{{\bar{x}}_{Muscle}+(0.0333\cdot {\bar{x}}_{Intestine})}$$

where TDI is the WHO TDI of 0.04 µg/kg, Weight is the weight of the adult consumer (assumed to be 60 kg in Table 3), ${\bar{x}}_{Muscle}$ and ${\bar{x}}_{Intestine}$ are the average concentrations of MCs in the muscle and tail (µg/kg), respectively, and 0.0333 is the ratio between the weight of the encapsulated intestine and the weight of the tail muscle based on measurements of 100 noble crayfish sampled in 2015.

The number of noble crayfish (z) that needed to be consumed to achieve the TDI was calculated using the formula:

(3) $$z=\frac{y}{4}$$

where y is weight of tail muscle (g) that needed to be consumed to achieve the TDI as calculated above, and 4 g is the average weight of a tail muscle.

## Figures and Tables

**Table 1:** PCR analyses on *Planktothrix*-cpcB-cpcA (PC-IGS) gene and the noble crayfish-specific cytochrome oxidase subunit I (COI) gene and MCs in stomach contents from *A. astacus* caught in 2015 and 2016 in L. Steinsfjorden and from four aquarium-cultured noble crayfish from Kasa crayfish farm at Hvaler.

Crayfish sample	Location	Month	Year	*Planktothrix* PC-IGS	*A. astacus* COI	MCs by ELISA (ng/g)
S74	L. Steinsfjorden	June	2015	+	+	362
S75	L. Steinsfjorden	June	2015	+	+	1310
S66	L. Steinsfjorden	June	2015	+	+	89
S62	L. Steinsfjorden	June	2015	+	+	292
S50	L. Steinsfjorden	June	2015	+	+	11^1^
S96	L. Steinsfjorden	Sept	2015	+	+	42
S91	L. Steinsfjorden	Sept	2015	−	+	140
S87	L. Steinsfjorden	Sept	2015	−	+	15856
S95	L. Steinsfjorden	Sept	2015	+	+	69
S97	L. Steinsfjorden	Sept	2015	+	+	22434
Ex1	L. Steinsfjorden	Oct	2016	−	+	1555
Ex2	L. Steinsfjorden	Oct	2016	−	+	25 ^1^
Ex3	L. Steinsfjorden	Oct	2016	−	+	3873
Ex4	L. Steinsfjorden	Oct	2016	−	+	345
Ex5	L. Steinsfjorden	Oct	2016	−	+	322
Ex6	L. Steinsfjorden	Oct	2016	−	+	258
Ex7	L. Steinsfjorden	Oct	2016	−	+	3982
Ex8	L. Steinsfjorden	Oct	2016	−	+	932
Ex9	L. Steinsfjorden	Oct	2016	−	+	17^1^
Ex10	L. Steinsfjorden	Oct	2016	−	+	125
1A1	Control (Kasa)		2016	−	+	n.a. ^2^
1B1	Control (Kasa)		2016	−	+	n.a. ^2^
4A1	Control (Kasa)		2016	−	+	n.a. ^2^
4B1	Control (Kasa)		2016	−	+	n.a. ^2^

^1^ values are between the limit of quantitation and the limit of detection in the ELISA. ^2^ n.a. = not analyzed, however pooled control crayfish from the same source in 2015 contained no detectable MCs.

**Table 2:** Summary of fixed effects included in the linear mixed-effects model describing the measured MC concentration (ng/g) in tissues. The model includes the significant factors, while it excludes the non-significant factors.

	Value	Std. error	*p*-value
Intercept (Hepatopancreas)	2.052	0.064	<0.001
Month (October_2015)	−0.203	0.151	0.180
Intestine	0.120	0.063	0.058
Muscle	−1.589	0.063	<0.001
Stomach	0.419	0.063	<0.001
Month (October_2015)*Intestine	−0.430	0.149	0.004
Month (October_2015)*Muscle	−0.339	0.149	0.023
Month (October_2015)*Stomach	0.048	0.149	0.747

**Table 3:** Calculated amounts of noble crayfish tail (by weight, and number of tails) with, and without, intestine, that would expose a consumer (60 kg) to the MC TDI based on mean levels found in edible tissues (tail muscle and intestine) in June–October 2015. A normal portion equals ~100 g crayfish muscle or ~25 crayfish tails.

Month	Weight of Tail Muscle (g)				Number of Noble Crayfish ^1^			
	w/intestine		w/o intestine		w/intestine		w/o intestine	
	Mean	SD	Mean	SD	Mean	SD	Mean	SD
June	165	152	484	417	41	38	121	104
July	175	186	451	822	44	46	113	206
August	155	83	629	621	39	21	157	155
September	76	35	283	280	19	9	71	70
October	462	264	766	349	115	66	192	87

^1^ Assuming 4 g of tail containing 133 mg intestine per noble crayfish.

## Supplementary Materials

The following are available online at https://www.mdpi.com/2072-6651/12/5/298/s1, Figure S1: Phytoplankton biomass and MC concentrations by Adda-ELISA in Lake Steinsfjorden for 2010–2016, Figure S2: Correlation between cyanobacterial biomass and microcystins in the water by the Adda-ELISA, Figure S3: Matrix studies of the control noble crayfish hepatopancreas, intestine and tail muscle tested in the multihapten-ELISA, Figure S4: Microcystins in crayfish tissue by multihapten-ELISA versus total length of noble crayfish, Figure S5: Microcystins in water by Adda-ELISA versus microcystins in the noble crayfish by multihapten-ELISA, Figure S6: Microcystins in edible tissues tail muscle and intestine in a meal in August 2015, Figure S7: Microcystins in edible tissues tail muscle and intestine in a meal in September 2015.
