# Supplementary material for: Microcystins in European Noble Crayfish Astacus astacus in Lake Steinsfjorden, a Planktothrix-Dominated Lake
Source: Toxins (Basel). 2020 May 5;12(5):298. doi: 10.3390/toxins12050298 (PMC7290910; doi:10.3390/toxins12050298)
Supplement: Supplementary file 1 [file toxins-12-00298-s001.pdf]

Supplementary Materials: Microcystins in European Noble Crayfish *Astacus astacus* in Lake Steinsfjorden, a *Planktothrix*-Dominated Lake

Ingunn Anita Samdal, David Allan Strand, Andreas Ballot, Johannes Christopher Rusch, Sigrid Haande, Kjersti Liv Eriksen Løvberg, Christopher Owen Miles and Trude Vrålstad

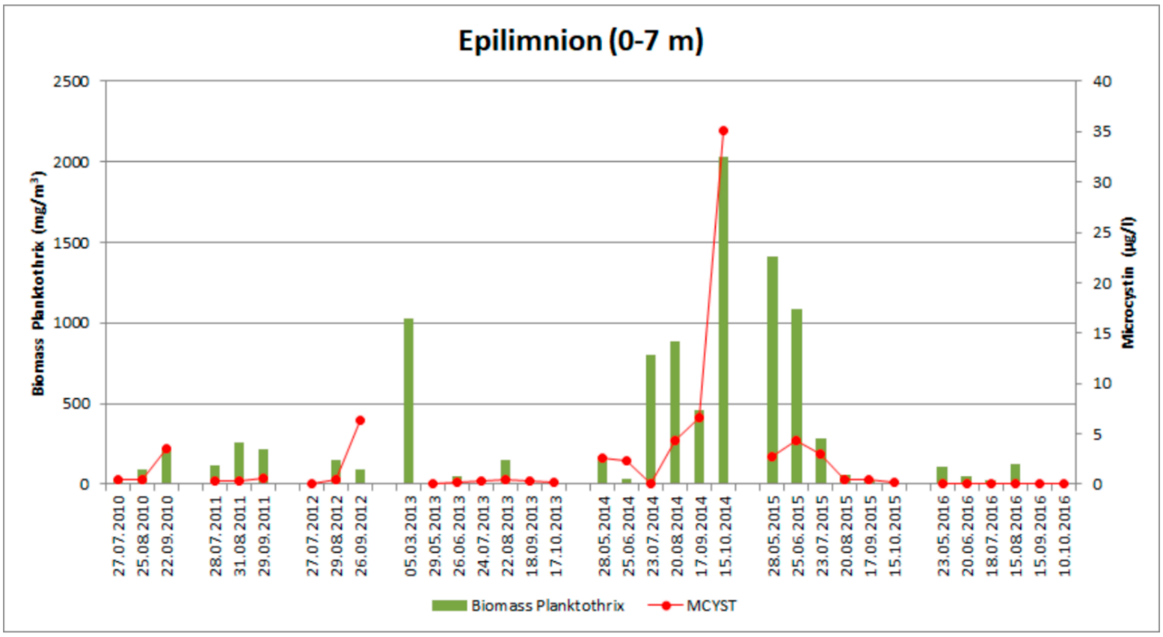

(**a**)

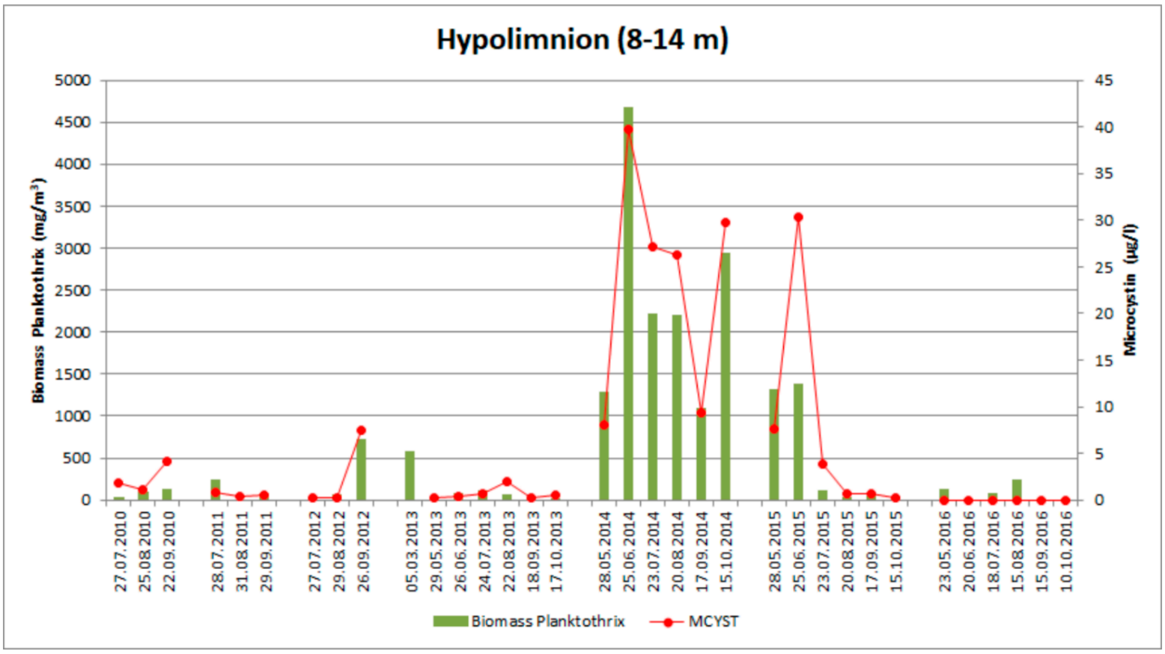

(**b**)

Figure S1. Phytoplankton biomass and microcystin concentrations by Adda-ELISA in Lake Steinsfjorden for 2010–2016 epilimnion (**a**) and hypolimnion (**b**).

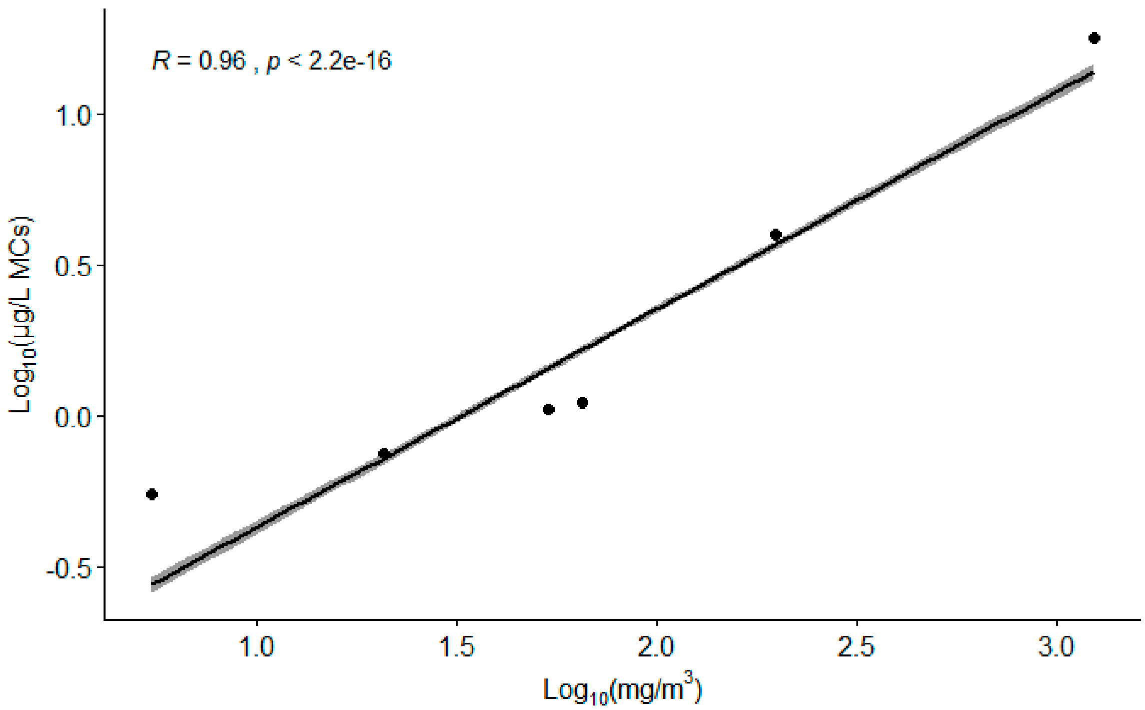

Figure S2. Correlation between cyanobacterial biomass and microcystins in the water by the Adda-ELISA.

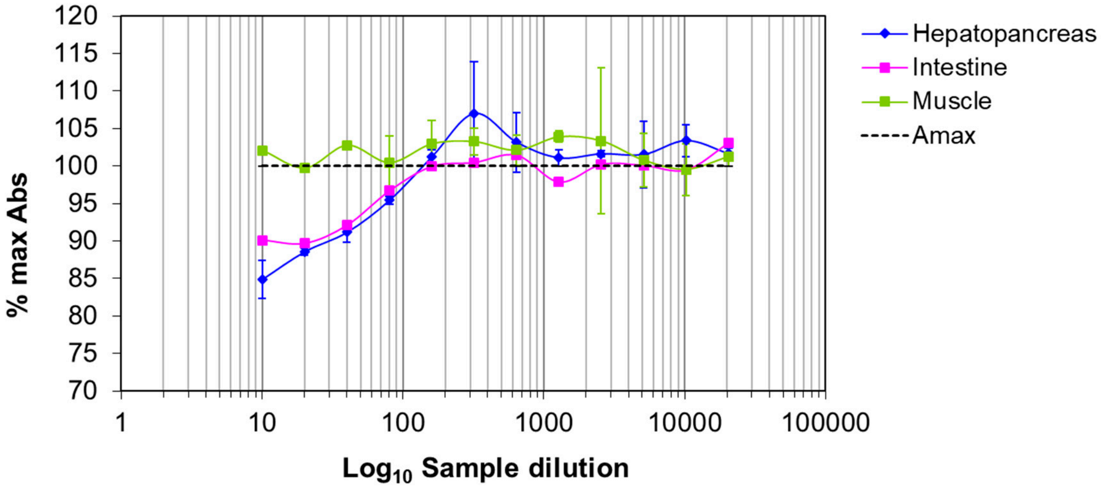

Figure S3. Effect of matrices from the control noble crayfish tissues hepatopancreas, intestine, and tail muscle, on the multihapten-ELISA. The tissues were tested with dilution series in duplicate on an ELISA plate with no MCs added, which corresponds to max absorbance on the plate. The experiment was repeated three times and mean values are shown. Max absorbance with sample buffer was set to 100% max absorbance (A_{max}). Matrix effects for tail muscle samples were not detectable with a 10-fold dilution of the sample matrix, whereas a ~100-fold dilution abolished the effect of the sample matrix on the ELISA for hepatopancreas and intestine.

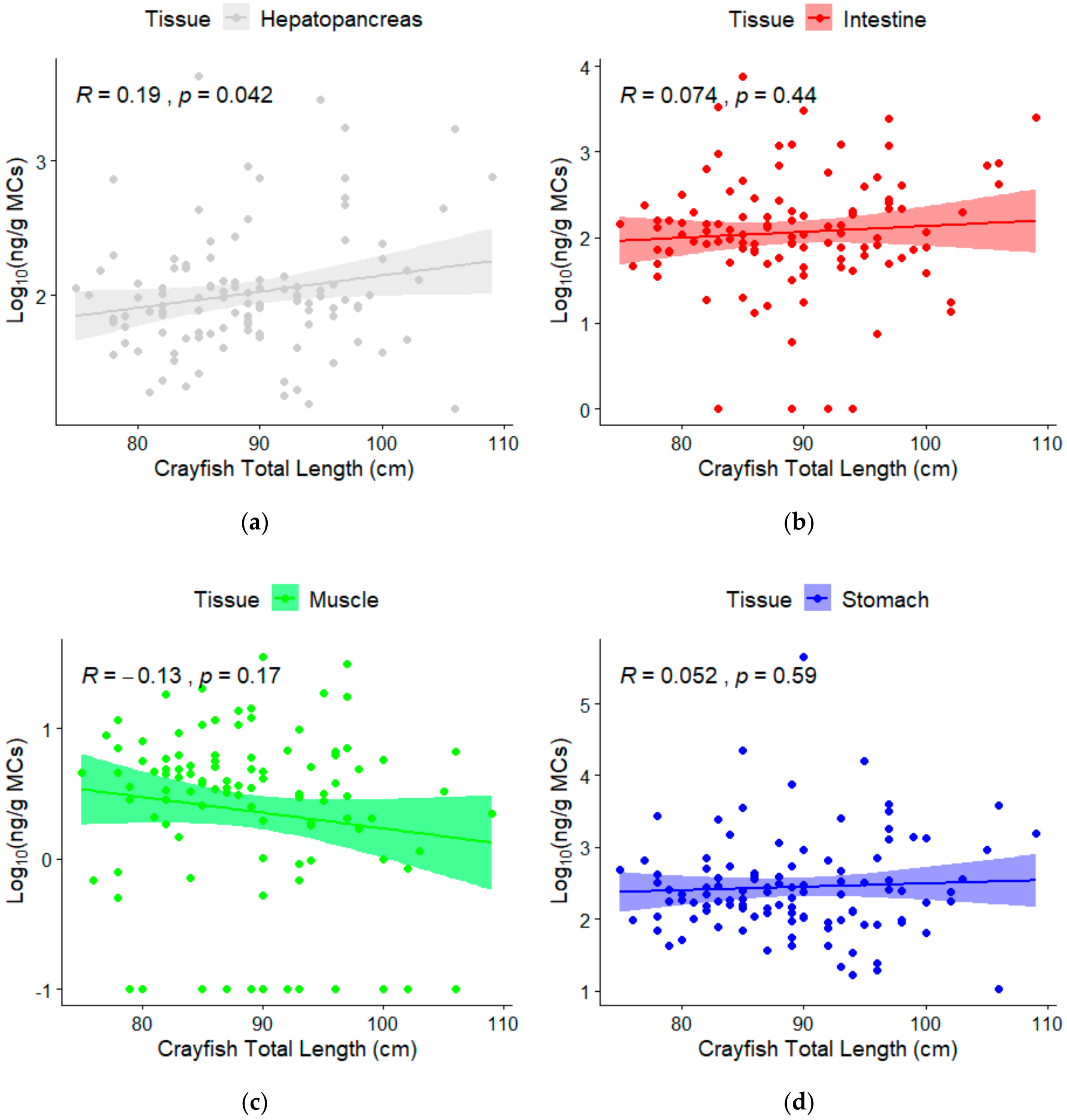

Figure S4. Microcystins in the four crayfish tissues by multihapten-ELISA versus the total length of noble crayfish; (**a**) hepatopancreas; (**b**) intestine; (**c**) tail muscle; (**d**) stomach, for 110 noble crayfish (n = 20 for each month, except for October 2016 where n = 10).

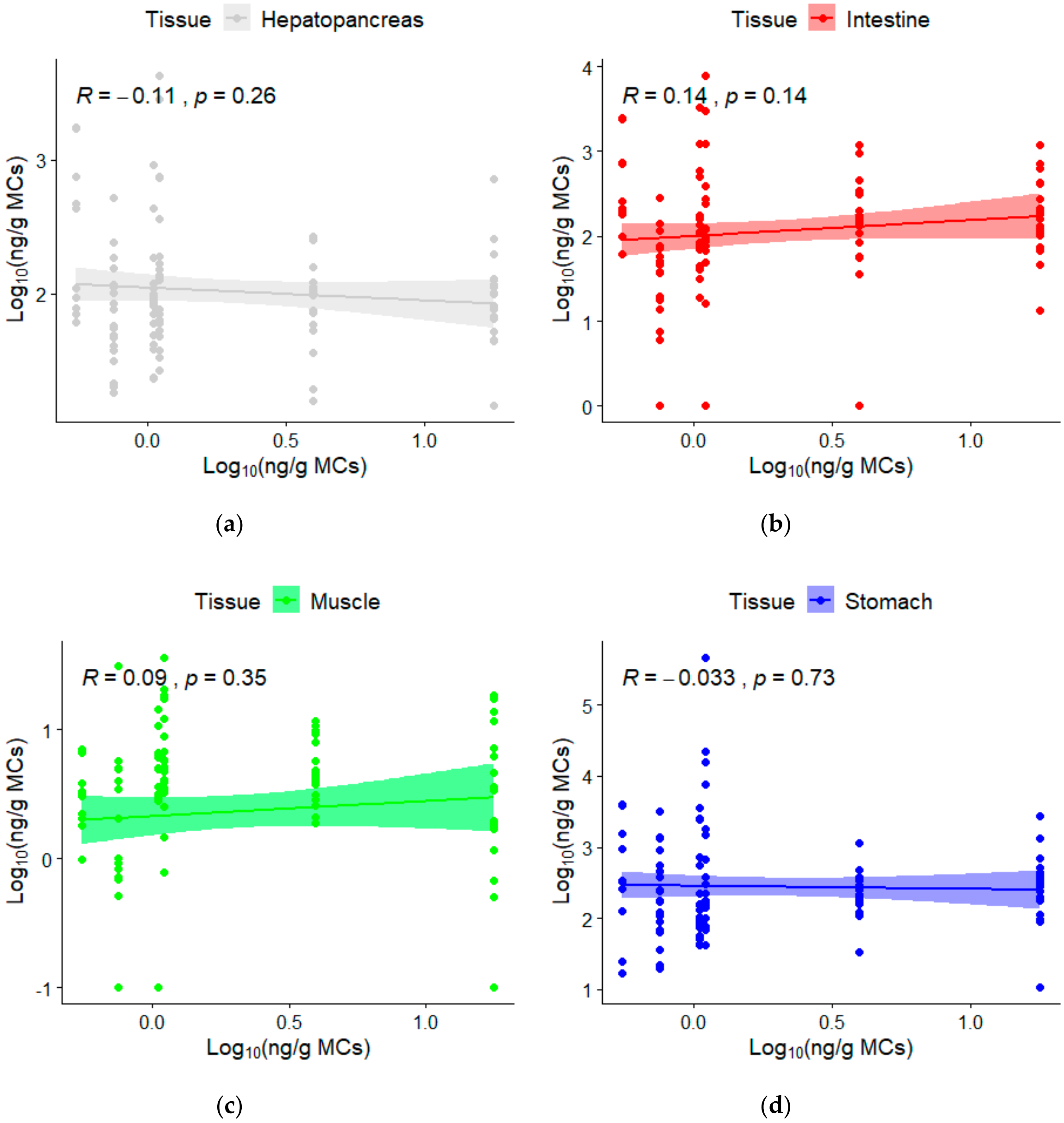

Figure S5. Microcystins in water from Lake Steinsfjorden by Adda-ELISA versus microcystins in the noble crayfish by multihapten-ELISA; (**a**) hepatopancreas; (**b**) intestine; (**c**) tail muscle; (**d**) stomach, for 110 noble crayfish (n = 20 for each month, except for October 2016 where n = 10).

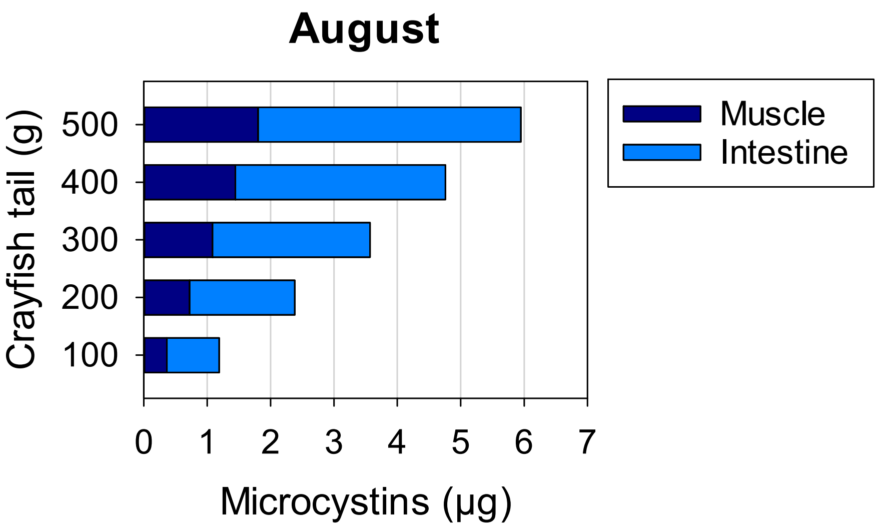

Figure S6. Contributions to total microcystins in edible tissues of noble crayfish (i.e., tail muscle and its encapsulated intestine) in a meal of crayfish harvested from Lake Steinsfjorden in August 2015, calculated for a range of meal sizes (100–500 g) from average observed microcystin concentrations.

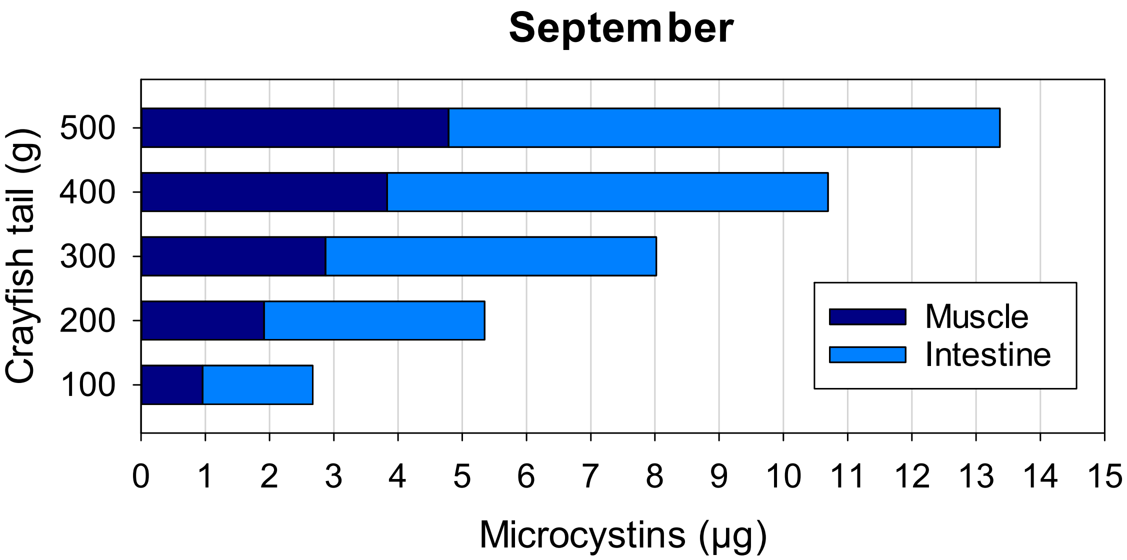

Figure S7. Contributions to total microcystins in edible tissues of noble crayfish (i.e., tail muscle and its encapsulated intestine) in a meal of crayfish harvested from Lake Steinsfjorden in September 2015, calculated for a range of meal sizes (100–500 g) from the average observed microcystin concentrations.
